# Seroprevalence of hepatitis B, C and D viral among hemodialysis patients in Tehran

**Published:** 2017-06

**Authors:** Davood Yadegarynia, Hossein Hatamai, Sara Rahmati Roodsari, Zahra Arab-Mazar

**Affiliations:** 1 Infectious Diseases and Tropical Medicine Research Center, Shahid Beheshti University of Medical Sciences, Tehran, Iran; 2 School of Public Health, Shahid Beheshti University of Medical Sciences, Tehran, Iran

**Keywords:** Seroprevalence, HBV, HCV, HDV, Hemodialysis

## Abstract

**Background and Objectives:**

Different studies show that the prevalence of hepatitis viruses in hemodialysis (HD) patients is much greater than general population. It is important to be aware of local prevalence data, in order to control infections and prevention of nosocomial transmission. The aim of this cross-sectional study was to investigate the seroprevalence of hepatitis B, C, and D viral infections among HD patients.

**Materials and Methods:**

During 2016, a cross-sectional study was conducted in Tehran, among 360 HD patients from 5 hemodialysis centers. All HBsAg positive subjects were screened for Hepatitis B surface Ag (HBsAg), Hepatitis C virus Ab (HCVAb) and Hepatitis D virus antibody (HDVAb), using specific enzyme linked immunoassay.

**Results:**

360 patients were involved including 213 males (59.17%) and 147 females (40.83%). The mean age in current study was 53.43 years. HBV positive (HBsAg positive) was found in 1.39% of patients, HCVAb in 3.06%, whereas no HDV positive patient was diagnosed. In HD, duration of 1–5 years has the highest frequency rate.

**Conclusion:**

Prevalence of HBV, HCV and HDV in hemodialysis patients seems low in Tehran province. Due to higher prevalence of HCV, it is recommended to check the patients for anti-HCV Ab before admission to the centers.

## INTRODUCTION

Viral infections are at increased risk of contraction, in patients undergoing dialysis treatment, and in particular HD due to the lack of cellular immunity, which increases their susceptibility to infection. However, this treatment helps to increase patients longevity, it also make them susceptible to some infection such as blood borne viruses. HBV and HCV infections are more frequent in HD patients compared to general population and are known to cause chronic liver disease (1, 2). This arises as a consequence of sharing dialysis machines or lack of inadequate infection control methods in hemodialysis centers (3).

More than 75% of HBV infected patients live in Asia and more than 8% of the population are chronic carriers (4). Prevalence rate of HBV in Iran is around 1.7–2.1 percent (5). In dialysis units, HBV transmission has been observed among patients to patient and patient to staff. In previous studies, the prevalence rate of HBV infection in HD patients ranged from 0% to 58% (6, 7). Viral hepatitis is complicating HD and it has been observed from the earliest days of this therapy. Routine screening of blood donor products, HBV vaccination and periodic measurement of anti-HBV antibodies help to decrease the prevalence rate of HBV in general population and HD in the past years (8).

Since HCV in most of infected patients are asymptomatic, it will be transmitted easily in the absence of standard infection control measures. Moreover, due to lack of an effective vaccine for HCV, it seems that HCV is the most common cause of chronic viral hepatitis in HD patients (9). The prevalence rate of HCV among HD patients is variable between different countries and between different studies (10) ranging from 3% in the Netherlands, Europe to more than 76% in Indonesia, Asia (11, 12). Also the prevalence rate varies among different dialysis centersin a single country (13–15). In the previous studies in Iran, the prevalence rate of HCV antibody has been reported 4.9%, inpatients of the central province, 5.5% in Shiraz (16), 9.55% in Rasht (17), 23.9% in Qazvin (18) and 13.2 % in Tehran (19).

Hepatitis D virus (HDV) is a small virus, requiring the concomitant presence of HBV for survival and pathogenicity (20). Approximately, 5% of the HBV patients are infected with HDV worldwide (21). In Iran, among HBsAg positive patients the 5.7–12.7 percent are infected with HDV (22). The HBV/HDV transmission is a major risk factor among HD patients. Different rates of HDV transmission in HD patients were reported in previous studies (23). It seems that it is important to know the prevalence of HDV infection in HD patient with HBV infection.

In this cross-sectional study, considering the importance of population study, we aimed to estimate the prevalence of HBV, HCV, and HDV infections in Tehran selected hemodialysis centers, in 2016.

## MATERIALS AND METHODS

This study was a cross-sectional study, conducted in five hemodialysis centers in Tehran province, in 2016. 360 patients, who were under HD treatment, were included in the study. The Ethical Committee of Shahid Beheshti University of Medical Sciences in Iran approved this study (ethics committee code: IR. SBMU. RETECH.REC.1395.462). All the enrolled participants were informed about study and written informed consent was obtained.

Demographic information such as gender, age, location of residence (urban vs. rural), education and marital status were collected from the patients (Table 1). Five mL of blood was withdrawn from each patient. The blood samples were centrifuged at 4000 rpm for 10 minutes and serum was separated and stored below −80°C for further use.

**Table 1.** Socio-demodraphic characteristics of hemodialysis patients and controls

**Variable**	**N**	**Percentage**
**Age (years)**		
>60	58	16.11%
40–60	132	36.67%
20–40	90	25%
<20	3	0.83%
**Marital Status**		
Single	25	6.94%
Married	335	93.05%
**Residence**		
Urban	262	72.77%
Rural	98	27.22%
**Educational level**		
Illiterate	100	27.77%
Primary School	190	52.77%
High School	57	15.83%
College and higher	13	3.6%
**Duration of hemodialysis (years)**		
>5	58	16.11%
1–5	213	59.17%
<1	89	24.72%

Serological markers of HBV (HBsAg: hepatitis B surface antigen, HBcAb: hepatitis B core antibody, and HBsAb: hepatitis B surface antibody) and HDV infection were tested in the present study via direct immunoenzymatic assay (24) of the sandwich type using commercial kits (Diapro, Italy). Samples found to be positive for HBsAb, were tested for anti-HDV antibody using immunoglobulin G (IgG) antibodies in the above-mentioned enzyme-linked immunosorbent assay (24) and also by using the competitive ELISA method. Anti-HCV antibodies was determined using ELISA (BIORAD, France).

Statistical analysis was performed using SPSS version 16 (SPSS Inc, Chicago, IL, USA).

## RESULTS

Total of 360 HD patients were participated in this study. 213 (59.17%) of patients were male and 147 (40. 83%) were female; the mean age of patients was 53.43 years. Based on serological assays, it was determined that five (1.39 %) patients were HBV positive (HBsAg-positive) 11 (3.06%) were HCV positive, whereas no HDV positive patients was found. The highest frequency for duration of dialysis is 1–5 years (Table 1).

## DISCUSSION

The present study was aimed to assess and determine seroprevalence of Hepatitis B, Hepatitis C and Hepatitis D viral infections among HD patients in Tehran, during 2016. In HD patients the prevalence of viral hepatitis and also the rate of mortality is much greater than general population. HD patients are recognized as one of the high-risk groups for hepatitis C virus (HCV) infection (25). Viral hepatitis is still a significant health problem especially in HD patients, particularly in the developing countries. Nowadays, early detection help us to better control and prevent the widespread presence of hepatitis in dialysis units.

Some investigators studied the prevalence of HBV, HCV and HDV in HD patients, but the results were varying in different population (26, 27). Alavian et al. reported that the prevalence of HBV among HD patients in Iran, was reduced from 3.8 % in 1999 to 2.6 in 2006 (2). The prevalence of HBV infection in the current study is low, compared with some similar studies (28–30).

Different studies confirmed that length of HD and use of shared machine was associated with high HBV prevalence in HD patients. In 2004, Sartor et al. reported that using shared devices in HD patients are associated with increased risk of viral infection (31). Patients were categorized in three groups based on the length of hemodialysis: Less than one year, between 1–5 years and longer than 5 years. Our study shows that the frequency of patients in 1–5 years category is higher compared to category of less than 1 year.

In present study, the rate of HCV infection in HD patients is comparable with other studies in other part of country. For example, in Tehran province, 1.9% of HD patients has positive HCVAb (32). In another study by Kheirabad, the rate of HCV in HD patients in Hormozgan province was 3.36% (33). It seems that we can conclude that Iran is one of the low prevalence countries for HCV, among HD patients.

Our findings revealed that in this study, performed in Tehran province, none of the patients were infected with HDV. The finding of this research study is compatible with recent studies in Iran (2, 29).

Among HD patients, especially HBs-Ag-positive patients, HDV infection become a major concern, while this virus can transmit at very high-serum titration (34). El Hady et al. demonstrated that HDV is much more frequently transmitted in HBV renal patients compared with HBV non-renal patients (35). HDV can cause chronic liver disease (36) and since an efficient proper treatment has not been found yet for HDV infection (37), prevention and early detection seems to be the best solution in order to limit the rate of transmission. As a result, designing an integrative protocol appears to play a vital role in lowering the morbidity and mortality among HBsAg-positive HD patients.

In conclusion, the prevalence of HBV, HCV and HDV infection in HD center of Tehran is moderate low. Educating people about HBV transmission risk factors and national vaccination help to decrease the prevalence rate among HD patients.
